# Prevalence and prevention of violence against children and adolescents in volunteer work: analysis of a sub-sample from a representative survey of Germany

**DOI:** 10.1186/s12889-025-23038-y

**Published:** 2025-05-27

**Authors:** Anna Eberhardt, Jörg M. Fegert, Elmar Brähler, Ulrike Hoffmann

**Affiliations:** 1 Department of Child and Adolescent Psychiatry, Psychosomatics and Psychotherapy, Ulm University Hospital, Steinhoevelstr. 5, 89075 Ulm, Germany; 2 German Center for Mental Health (DZPG), partner site Ulm, Steinhoevelstr. 5, 89075 Ulm, Germany; 3 Department for Psychosomatic Medicine and Psychotherapy, University Medical Center, Johannes Gutenberg University of Mainz, Untere Zahlbacher Str. 8, 55131 Mainz, Germany; 4 Department of Medical Psychology and Medical Sociology, University of Leipzig, Philipp-Rosenthal-Str. 55, 04103 Leipzig, Germany

**Keywords:** Violence, Prevention and control, Voluntary workers, Child abuse, Child welfare

## Abstract

**Background:**

Violence against children and adolescents, including physical, emotional, and sexual maltreatment as well as neglect, is a global issue that has long-term consequences. This violence can occur in various institutions, including children and youth clubs (CACs), where proximity to children may be exploited. Although some studies have documented violence in CACs (VCAC), its full extent often remains unrecognized. Despite ongoing prevention efforts, data on the successful implementation of those efforts are lacking. Since protection of VCAC and support of those affected is crucial, this study aimed to determine VCAC prevalence rates, the frequency of measures for protecting children and adolescents from VCAC as well as supporting them, and their relationship to feelings of safety in a German sample.

**Methods:**

From October 2023 to March 2024, a representative sample of 2,513 people aged 16 years and older in Germany was surveyed. The survey collected sociodemographic information and details on voluntary activities in CACs, focusing on experiences with VCAC and safeguarding measures. Descriptive statistics were used to analyze the sample and VCAC prevalence, regressions were used to examine perceptions of protective measures and subjective well-being. The study adhered to ethical guidelines and was approved by the University of Leipzig’s Ethics Committee.

**Results:**

Among 954 respondents which were active in CACs 819 were looked after as child/adolescent, of those 8.42% experienced VCAC − 6.22% emotional, 5.74% physical, and 1.10% sexual violence. The most common protective measures included discussions about violence (42.49%) and complaint procedures (29.06%). Experience with VCAC were negative associated with perceived protective measures and subjective well-being. Subjective well-being was positive associated with VCAC taken seriously in the CACs.

**Conclusions:**

These findings highlight the importance of CACs for the development of children and adolescents. However, 7% of people involved in CACs experience at least one form of VCAC, which indicates that there is still room for improvement in the protection of children and adolescents. Furthermore, existing prevention measures are often still insufficiently known, which points to a communication gap. This finding highlights the importance of mandatory safeguarding measures and better training for volunteers to effectively safeguard children and adolescents in CACs.

**Supplementary Information:**

The online version contains supplementary material available at 10.1186/s12889-025-23038-y.

## Background

Violence against children and adolescents is a far-reaching and serious societal problem worldwide. This includes physical, emotional, or sexual violence as well as neglect, which impairs the well-being and development of children and adolescents. Physical violence is defined as the targeted use of violence against the child/adolescent that leads to, or can lead to, physical injury. Emotional violence is any intentional behavior that conveys to the child/adolescent that they are worthless, flawed, unloved, unwanted, or useless and thus (potentially) causes psychological or emotional harm. Sexual violence includes any sexual act carried out or attempted with or without direct sexual contact with the child/adolescent. In the case of neglect, a distinction is made between failure by the guardian to provide for the basic needs of the child/adolescent and failure to supervise as a lack of ensuring the child’s safety [1].

Meta-analyses have shown that the prevalence of various forms of child maltreatment is high worldwide. The prevalence for the different forms of maltreatment ranged from 0.3% (emotional and physical violence) to 0.4 (sexual violence) in studies using third party reports and 12.7% (sexual violence), 22.6% (physical abuse) and 36.3% (emotional abuse) in studies using self-report measures [2–4]. While the discussion on child maltreatment has long focused primarily on adults as perpetrators, various forms of violence among peers, such as physical and emotional violence and bullying, have also become increasingly important in recent years. A recent WHO survey on the prevalence in various countries in Europe, Central Asia and Canada shows that around 11% of children and adolescents are affected by bullying, 12.5% by cyberbullying and 10% by physical confrontations [5].

Overall, studies have repeatedly revealed that experiencing violence has an increased risk of short-term and long-term health and social consequences [6–9]. International findings also highlight a correlation between the different types of maltreatment. The presence of one form of maltreatment increases the likelihood that the affected person also suffers from other forms of maltreatment (multiple victimization) [10–13]. A 2020 systematic review by Carr and colleagues that analyzed 111 reviews showed that child maltreatment is linked to numerous negative consequences. Twenty systematic reviews and meta-analyses found significant associations between maltreatment and physical health problems, including neurological, muscular, cardiovascular, and gastrointestinal diseases, as well as impaired immune function. A strong association between maltreatment and mental illness was also found in 45 papers. Compared to children and adolescents who have not experienced maltreatment, those affected have a twice as high risk of developing depression and have higher rates of anxiety disorders, PTSD, bipolar disorders and psychosis. Furthermore, 55 papers show that maltreatment causes widespread psychosocial impairments, such as cognitive deficits, language delays, insecure attachment, school problems, antisocial behavior, sexual aggression, risky sexual behavior, and parenting problems. The results also indicate that both the type and intensity of maltreatment can influence the consequences and that multiple exposures can have particularly serious effects on health [14]. Furthermore a current study have shown that maltreatment experiences (by adults and/or peers) in institutions during childhood and/or adolescence also increases the risk for mental and physical illness and restrictions in health-related quality of life, like impairment in mobility, self-care, usual activities and activities of daily life, as well as pain/discomfort and anxiety/depression [15]. The significance of child maltreatment for the public health sector is therefore particularly high.

We know that most violence against children and adolescents takes place in the family context, but studies indicate that also institutions that work with or care for children and adolescents can become scenes of child maltreatment [16, 17]. Among these contexts, child and adolescent clubs (CACs) play an important role. For Germany CACs are organizations or programs often run by volunteers, sometimes also by full-time staff, that care for and work with children/adolescents to promote their development and well-being and to help them develop into responsible and active members of society. Examples include sports clubs, youth groups, church organizations and vacation camps. Alongside home and school, CACs are often particularly important developmental spaces where children and adolescents can test themselves in a protected environment, pursue their interests and form social bonds [18]. Furthermore, CACs are also important as places to support those affected by violence outside the CACs. They can offer a safe environment in which those affected by violence can seek guidance and trustful contact persons [19]. According to the 2019 German Survey on Volunteering, 39.7% of Germans 14 years of age and older volunteer, half of whom work with children and adolescents as trainers, youth leaders or mentors, for example [20, 21]. Voluntary activities include hereby in addition to looking after children and adolescents a variety of tasks, such as practical and organizational activities, helping, advising, and mentoring tasks [21]. The intensity of the work ranges from merely selective assignments, to the assumption of individual areas of responsibility, to the independent management and administration of programs [22]. Among the approximately 15.1 million children and adolescents in Germany, six million were supervised in youth associations [23], and 7.6 Million in sports clubs [24]. This nature of volunteer work enables and requires special trust between children/adolescents and volunteers. However, CACs also present several risk factors for the occurrence of violence in CACs (VCAC), such as a lack of expertise and awareness regarding (sexualized) violence, the absence of opportunities for sanctions, diffuse and unclear structures and responsibilities, and close relationship structures between volunteers and children/adolescents, which can be manipulated and abused. The physical and emotional closeness involved in working with children and adolescents can also be a risk factor for VCAC [25]. However, there is currently still a lack of comprehensive data on the prevalence of VCAC in Germany.

Research has already documented some cases of VCAC, which raises serious concerns about the safety and well-being of children and adolescents in CACs. The “SicherImSport” (Translation by the authors: Safe in Sports) study investigated how frequently violence occurs in sports clubs in Germany. For this purpose, 4,367 athletes with an average age of 41.8 years (46.3% women, 52.6% men and 0.5% stated a different gender), of whom 688 were amateur athletes, were asked about their experiences of violence in sport. More than half (53%) of amateur athletes surveyed had experienced violence. Emotional violence (44%), sexualized violence without physical contact (19%) and physical violence (18%) were reported most frequently by amateur athletes. The most common perpetrators (up to 71%, depending on the type of violence) were main coaches, followed by athletes in the same training group (up to 62%) and co-coaches (up to 57%). The majority of those affected (69%) remained silent about their experiences and did not inform the organizations concerned [26].

Furthermore, many perpetrators of sexual violence specifically seek proximity to children and adolescents in volunteer work because these often offer lower barriers to entry. According to perpetrator profiles, these individuals often present themselves as socially adapted and committed members, making it difficult to identify them early and take appropriate action [27]. The World Health Organization (WHO) also assumes that 90% of cases of child maltreatment in institutions go unrecognized [28]. One of the reasons for this is that contact people often have too little knowledge about symptoms and ways of dealing with suspected cases. For some years, efforts have been made in Germany to establish safeguarding measures as a set of elements to prevent violence and support those affected, for example, risk analysis, intervention plans, staff training and codes of conduct. To date, there is a lack of data on the extent to which these elements or safeguarding measures as a whole have been implemented successfully in volunteer groups [25].

In summary, comprehensive data on VCAC and prevention strategies for CACs are still lacking in Germany and internationally. Given that approximately half of the volunteers in Germany regularly work with children and adolescents, it is crucial to gather more data on the prevalence of VCAC, prevention strategies and their influence on health and well-being of children and adolescents. Having shown the enormous impact that experiencing child maltreatment has on health, it is equally important to examine the extent to which prevention measures can increase the well-being of children and adolescents. The present study aims to determine the prevalence rates of VCAC, the frequency of protective measures, and their relationship to feelings of safety among CACs and analyses therefore the following research questions.

- What are the current prevalence rates of emotional, physical, and sexual VCAC in Germany?
- How frequently are measures for protecting children and adolescents from VCAC and supporting those affected implemented in CACs in Germany?
- How is the perceived sense of safety among children and adolescents associated with the presence of measures for protecting children and adolescents from VCAC?

## Methods

### Sampling

A total of 2,513 people aged 16 and older were interviewed between 10/2023 and 3/2024 as a representative sample of the German population. Qualified interviewers were engaged for the survey, who received written instructions for this project with information on the survey procedure and specific explanations of the questionnaire content. The interviews were conducted in person at the respondents’ homes. In the first step, research staff collected sociodemographic information in an interview format. Then, the researcher handed out a copy of the questionnaire and a sealable envelope and stayed nearby to answer possible questions about the questionnaire. Approximately 53,000 geographic areas were defined in Germany, containing at least 350 households. These were divided into 1,500 regional segments, and 128 networks were drawn proportionally to the distribution of private households. Within the regional areas, households were selected using the random route method. The target person in the household was determined using the “Kish-Selection-Grid”, as a method for the random selection of respondents in households with several people [29]. The data were weighted to ensure representativeness. The design weighting corrected the probability of selection within the households, and an adjustment weighting compensated for distortions due to nonresponses. The distributions of age, gender and place of residence were adjusted to the 2022 micro census data. At the end of the survey phase, the marginal distributions of the main survey were compared with those of the supplemented subsample, and possible distortions were balanced out by weighting to ensure the representativeness of the results. As the sample was subdivided according to “activity in CAC”, the composition of the sub-sample was subsequently no longer representative.

### Data collection

An overall questionnaire consisting of two parts was developed. In the first part, the interviewer collected sociodemographic data on the target person and the household at the date of the survey in accordance with the demographic standards of the Federal Statistical Office. In the second part, the questionnaire was completed independently, with the interviewer providing support where necessary.

The sociodemographic questions used for this study included age, gender, and monthly net income per household (at the date of the survey). Data were collected in a questionnaire developed for this study on current or past activity as a child and/or adolescent as well as an adult in a CAC. The survey asked about membership in sports clubs, music clubs, youth political associations (e.g., Young Socialists of the German Democratic Republic, youth organizations of political parties, Young European Federalists), community clubs with a societal or charitable focus (e.g., nature conservation youth or workers’ welfare youth organizations), social/rescue associations (e.g., German Red Cross, volunteer fire department), and church associations (e.g., ministrants). All others could select the option “Other” (see Additional file 1). For the collection of subjective well-being data at the date of the survey, the World Health Organization-Five Well-Being Index (WHO-5) in German was used. The WHO-5 is a short, internationally recognized questionnaire for measuring subjective mental well-being. It consists of five positively formulated statements that are rated on a scale that ranges from 0 (never) to 5 (always). For Germany, the WHO-5 Well-Being Index has excellent internal consistency (Cronbach’s alpha = 0.92), high test-retest reliability (r_tt_ = 0.87) and a single-factor structure with a variance explanation of 75.53% and is therefore a reliable measure of general well-being [30]. In the present sample, the WHO-5 showed excellent internal consistency (Cronbach’s alpha = 0.94). The exploratory factor analysis revealed a unidimensional structure with a variance explanation of 81.44%.

The focus of the survey was on the experiences of the respondents who were affected by VCAC and protective measures taken by CACs. Because there is currently no standardized measuring instrument for VCAC, the survey therefore collected the forms of maltreatment defined by the Centers for Disease Control and Prevention (CDC) [1] with a dichotomous variable, here is no distinction made as to whether the VCAC is practiced by adults or peers. VCAC experiences were only surveyed for participants who were active in a CACs as a child/adolescent. The only form of maltreatment that was not surveyed in CACs was neglect because the perception of neglect by those affected, especially in the volunteer sector, is considered complex and would have gone beyond the scope of the questionnaire. However, neglect was included in the question about experiences of maltreatment in the family. The prevention activities were based on the safeguarding measures that were recommended by the “Round Table on Child Sexual Abuse in Relationships of Dependency and Power,” which dealt with the abuse scandal in 2010, in the course of which sexual abuse of children and adolescents in church and state institutions in Germany was uncovered [31]. In summary, 15 elements were surveyed. The elements “(Sexual) educational concept,” “Extended certificate of good conduct,” “Further training on (sexual) violence for volunteers/employees,” and “Intervention plan for dealing with (sexual) violence” were collected only from participants who were active in CACs as adults because it is assumed that children and adolescents do not actively perceive these.

In addition, data were collected on how respectful and friendly the interaction between the individual members of the association was on an end-point scale that ranged from 1 (very less respectful and friendly) to 6 (very respectful and friendly) and were treated quasi-metric. These items asked about a sense of safety from violence, respectful behavior of children, adolescents, and trainers/leaders in the CACs, and the handling and tolerance of cases of any form of violence in the CACs.

### Data analysis

Descriptive statistics were used to characterize the sample, the prevalence of VCAC, the frequency of elements of safeguarding measures, and the sense of safety in the CAC. the responses to the items of the WHO-5 were summed to form an index (minimum: 0; maximum: 25), whereby higher values indicate better subjective well-being. Furthermore, the experience of the different forms of VCAC was supplemented with a dichotomous variable on the experience of VCAC, and all mentioned elements of safeguarding measures in the CACs were added to a metric variable that had a minimum value of 0 and a maximum value of 15. A linear regression was used to analyze factors related to the perception of the number of protective measures in the CACs with the predictors Age, Gender, Activity in Sports/Music/Youth/Political/Community/Social/Rescue/Church CACs (each as a dichotomous variable), experienced VCAC and sense of safety in the CACs (score on 5 items: minimum: 5; maximum: 30). Another linear regression was used to calculate the determinants influencing subjective well-being at the time of the survey, with the predictors Age, Gender, Equivalent income, Experienced VCAC and VCAC being taken seriously in CACs. All analyses were performed using SPSS version 29 [32]. The level of statistical significance was set at *p* < 0.05 [33].

### Ethics & consent

The participants were given an explanation of the study’s purpose. Individuals who agreed to participate were informed about the study, and informed consent was obtained. In the case of minors, informed consent was given by the participants themselves, with the consent of their guardians being obtained.

The study was conducted in accordance with the Declaration of Helsinki and complied with the ethical guidelines of the International Code of Marketing and Social Research Practice of the International Chamber of Commerce and the European Association for Opinion and Marketing Research. The Ethics Committee of the Medical Faculty of the University of Leipzig approved the study.

## Results

### Description of the sample

Of the 2,513 respondents, 1,299 (51.69%) were male, 1,212 (48.22%) were female, and two persons (0.07%) was of a diverse gender. On average, the respondents were 49.56 years old (standard deviation (SD): 17.42) and had a net income of 2,240.71 € (SD: 1,077.89). 954 (37.96%) were active members of one or more clubs whose purpose included working with children and/or adolescents (Table 1).

**Table 1:** Sample characteristics (*N* = 2,513)

	Overall Sample		Activity in CAC as child/adolescent or adult (*n* = 954)		Activity in CACs as child/adolescent (*n* = 819)	
	Mean	SD	Mean	SD	Mean	SD
Age (years)	49.56	17.42	49.38	17.16	48,30	17,22
Equivalent income per month (€)	2,240.71	1077.89	2,340.70	1,098.30	2,330.18	1,097.23
Subjective well-being^1^ (Min: 0; Max: 25)	16.54	18.00	17.02	5.32	17.02	5.26
	n	% of 2,513	n	% of 954	n	% of 819
Gender Female	1,299	51.69	456	47.79	397	48.47
Male	1,212	48.22	497	52.09	421	51.40
Diverse	2	0.07	1	0.10	1	0.12
Experience of Child Maltreatment in the Family	578	23.00	232	24.31	198	24.17

SD = Standard deviation; ^1^assessed using World Health Organization-Five Well-Being Index

**Fig. 1:**
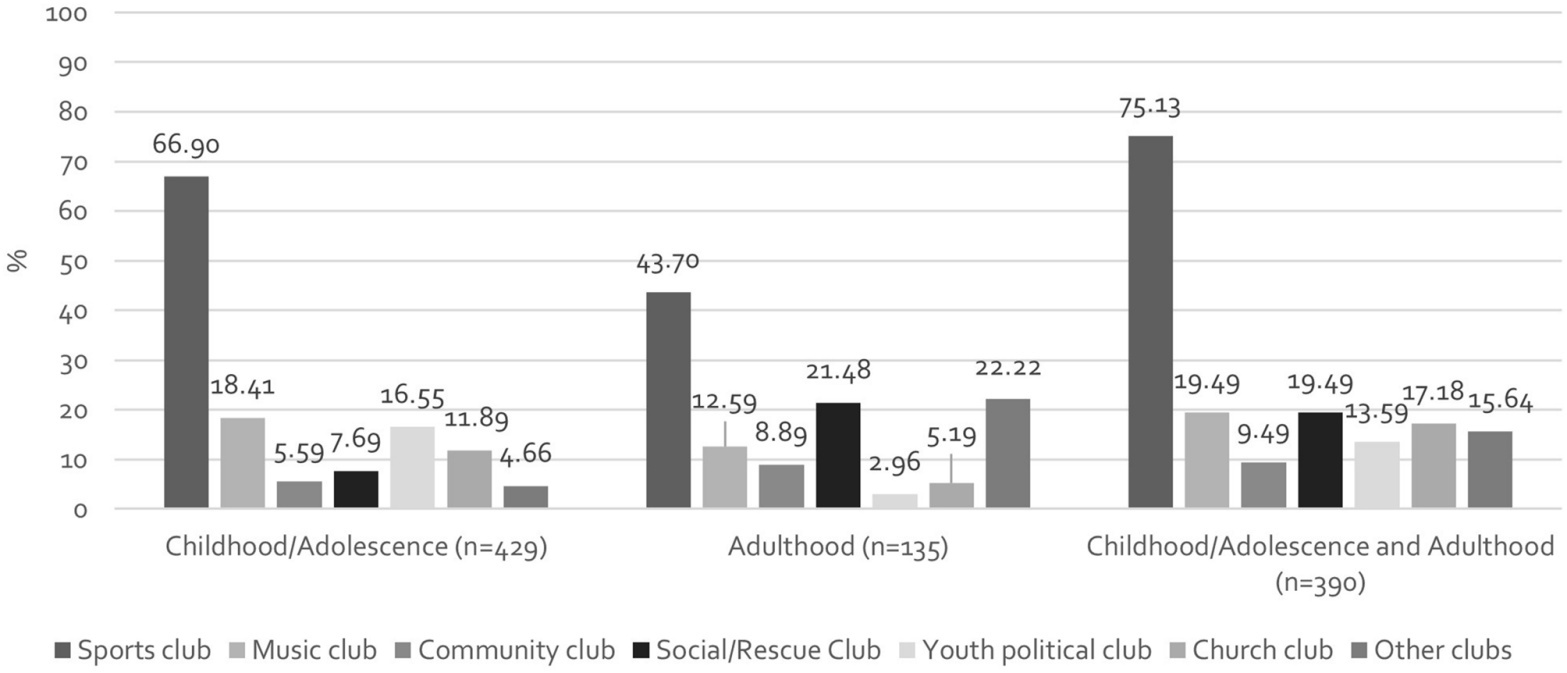
Activity in different types of CACs according to life stage (multiple answers possible), *N* = 954

Of the 954 respondents who were active members in CACs a total of 135 people (14.15%) were active only as adults, 429 (44.96%) were active only as children/adolescents, and 390 (40.88%) were active both as children/adolescents and adults. Figure 1 shows the activities of the different types of clubs according to life stage. In the case of memberships that were active only during childhood and adolescence, sports (66.70%), music (18.41%), and youth political clubs (16.55%) were the most common. The respondents who were only active in CACs as adults, were also particularly active in sports (43.7%) and social or rescue clubs (21.48%). People who were active as volunteers both as children/adolescents and as adults were, in addition to sports clubs (75.13%), particularly active in music clubs (19.49%), social and rescue clubs (19.49%), and church clubs (17.18%). Overall, adults who were already active in clubs as children/adolescents were more often involved in volunteer work with children and adolescents.

### Prevalence of VCAC

Among the 819 participants who were voluntarily active in clubs working with children and/or adolescents, 8.42% (*n* = 69) reported experiencing VCAC themselves in their CACs (for details, see Table 2).

**Table 2:** Prevalence of VCAC in childhood/adolescence by gender and type of abuse/violence (*n* = 819)

	Total		Emotional VCAC		Physical VCAC		Sexual VCAC	
	n	%	n	%	n	%	n	%
Male (*n* = 421)	43	10.21	28	6.65	32	7.60	1	0.24
Female (*n* = 397)	26	6.55	23	5.79	15	3.78	8	2.02
Total (*n* = 819^1^)	69	8.42	51	6.22	47	5.74	9	1.10

^1^ A participant of diverse gender did not mention violence in the volunteer context

Overall, 54.1% (*n* = 33) of participants who reported any form of VCAC reported only one type, 45.9% (*n* = 28) reported two types, and 6 (9.8%) participants reported all three types. In particular, girls suffered from all three forms of VCAC; boys often suffered from emotional as well as physical VCAC but not from sexual VCAC (Table 3).

**Table 3:** Number of experienced types of VCAC by gender (*n* = 67^1^)

	Number of experienced forms of VCAC		
	1	2	3
Male	23	19	0
Female	10	9	6
**Total**	**33**	**28**	**6**

^**1**^ Two participants mentioned the experience of VCAC, but not the specific form

### Perception of safety and protection measures in CACs

With respect to the protective measures for violence in CACs, respondents frequently perceived the addressing of violence (42.49%) as a child/adolescent, whereas only 22.48% of the adults had done so, followed by opportunities to complain (29.06%) and other information concerning (sexual) violence (26.13%). Adults in CACs were most likely to perceive the participation opportunities for children and adolescents (36.95%), which was only perceived by 8.91% of respondents who were active in CACs in childhood and/or adolescence, a code of conduct (33.33%) and opportunities to complain (31.24%). While 15.05% of adults, were aware of prevention programs against (sexual) violence, only 4.88% of the participants active in CACs as child/adolescent mentioned this. Furthermore, only 4.38% of adults reported further training, and 5.52% reported that their CACs had an intervention plan in the event of (sexual) violence (Table 4). In addition, only 7.5% of respondents who were active as children and/or adolescents in a CAC, and 10.7% of adults, reported the development of safeguarding measures in their volunteer organization.

**Table 4:** Frequencies of protective measures in the CACs observed

Protective measures observed in the CAC	Member of CACs in Childhood/Adolescence (*n* = 819)		Member of CACs in Adulthood (*n* = 525)	
	*n*	%	*n*	%
Participation opportunities for children/adolescents	73	8.91	194	36.95
Opportunities for complaints	238	29.06	164	31.24
Naming a person who can be approached if (sexual) violence is experienced or suspected	143	17.46	107	20.38
Code of conduct for dealing with each other in the club	200	24.42	175	33.33
Addressing misconduct in the context of (sexual) violence by youth leaders/trainers	121	14.77	73	13.90
Punishment of misconduct in the context of (sexual) violence by youth leaders/trainers	73	8.91	56	10.67
Implementation of prevention programs	40	4.88	79	15.05
Information (e.g., posters. notices. flyers) on (sexual) violence	214	26.13	150	28.57
Addressing the topic of (sexual) violence by youth leaders/trainers	348	42.49	118	22.48
Development of safeguarding measures	61	7.45	56	10.67
(Sexual) educational concept for working with children/adolescents^1^	-	-	22	4.19
Requesting an extended certificate of good conduct from all volunteers/employees^1^	-	-	49	9.33
Further training on (sexual) violence for all volunteers/employees^1^	-	-	23	4.38
Intervention plan for dealing with (sexual) violence^1^	-	-	29	5.52
Other	234	28.57	141	26.86

^1^ Not surveyed for participants who were active in CACs only as a child/adolescent

Table 5 shows that most of the respondents felt relatively safe in their CACs regarding VCAC, both as children and adolescents and as adults, and cases of VCAC were mostly taken seriously and were not tolerated. However, the interaction between children and adolescents was not always described as respectful.

**Table 5:** Sense of safety among children/adolescents and adults in CACs surveyed on a six-level endpoint scale

Item	*n*	Mean	SD	Median	IQR
I feel/felt safe from VCAC in my CAC.	814	5.59	1.002	6	0
The youth leaders/trainers treat/treated all children/adolescents with respect.	812	5.51	0.986	6	1
The children/adolescents treat/treated each other respectfully.	813	4.94	1.171	5	2
Cases of VCAC are/were taken seriously in my CAC.	791	5.46	1.067	6	1
VCAC of any kind is/was not tolerated in my CAC.	806	5.54	1.007	6	0

SD: Standard deviation; IQE: Interquartile range

The linear regression shows how the perception of the number of protective measures in CACs is associated. The adjusted R² for the overall regression model was 0.05, indicating a low variance resolution, according to Cohen [34]. The model is significant at the 0.1% level. Age at the date of the survey older than 60 years, membership in a music, social/rescue or youth political club, and experienced VCAC were statistically significantly associated with the number of perceived protective measures. Younger respondents (< 40 years) perceived significantly more protective measures than the oldest age group (> 60 years) (B = 0.514, *p* < 0.001), followed by 40–60-year-olds, who also show higher values (B = 0.343, *p* = 0.003). This indicates that the perception of protective measures decreases with increasing age at the time of the survey. If one was a member of a music, youth political or social/rescue club, the number of perceived protective measures in the CACs rose (music club by 0.26 times, youth political club by 0.55 times, social/rescue club by 0.43 times). In addition, the number of perceived protective measures declined by a factor of 0.52 if one had not experienced VCAC in the CACs (Table 6).

**Table 6:** Associations between perceived protective measures, age, gender, activity in different forms of CAC, experienced VCAC, and sense of safety in CAC: results from a linear regression analysis (*n* = 780)

	Factor	B	*p*	95% CI	
Adjusted R^2^ = 0.05 F (11, 769) = 4.496 *p* < 0.001***	Constant	1.877	< 0.001***	1.133	2.621
	Age at the date of the survey < 40^1^	0.514	< 0.001***	0.281	0.748
	Age at the date of the survey 40–60^1^	0.343	0.003**	0.119	0.568
	Gender^2^ (female)	0.025	0.792	-0.158	0.207
	Sports club^3^	0.132	0.234	-0.085	0.348
	Music club^3^	0.26	0.035*	0.019	0.5
	Youth political club^3^	0.551	< 0.001***	0.29	0.812
	Community club^3^	0.27	0.124	-0.074	0.614
	Social/rescue club^3^	0.425	0.002**	0.157	0.694
	Church club^3^	0.048	0.718	-0.214	0.31
	Experienced VCAC (no)^4^	-0.515	0.005**	-0.877	-0.153
	Sense of safety in CACs (Score) ^#^	0.023	0.056	-0.001	0.046

CI = Confidence Interval; ^#^ Continuous Variable; * Significant at the 5% level; ** Significant at the 1% level; *** Significant at the 0.1% level; ^1^ Reference category: age at the date of the survey > 60; ^2^ Reference category: male; ^3^ Reference category: no activity in this CAC; ^4^ Reference category: experienced VCAC

### Subjective wellbeing and the experience of VCAC

Table 7 shows the association between subjective well-being and experiences in CACs. The model explains 24% of the variance and is highly significant (*p* < 0.001). People under 40 have a significantly higher subjective well-being than people over 60 (B = 1.737), while the difference between 40- to 60-year-olds and the oldest group is not statistically significant (B = 0.433). Furthermore, for every euro more of equalized income per month, well-being increased by a minimal amount. The assessment of the handling of VCAC cases in the CACs shows that an increase in cases being taken seriously is associated with an improvement in subjective well-being of 0.808 points. People who had not experienced maltreatment in the family reported significantly greater subjective well-being, 3.8 times greater than that reported by those who had experienced maltreatment. This effect is the largest in the model and has a very high significance level, which strongly emphasizes the importance of family experiences for well-being.

**Table 7:** Associations between subjective well-being, age, gender, experienced VCAC, taking VCAC seriously in the CAC, and experienced maltreatment in the family: results from a linear regression analysis (*n* = 778)

	Factor	B	*p*	95% CI	
Adjusted R^2^ = 0.24 F(7, 770) = 35.60 p = < 0.001***	Constant	0.788	0.57	-1.929	3.504
	Age at the date of the survey < 40^1^	1.737	< 0.001***	0.896	2.578
	Age at the date of the survey 40–60^1^	0.433	0.302	-0.391	1.258
	Gender^2^ (female)	-0.08	0.809	-0.732	0.571
	Equivalent income per month at the date of the survey (€)^#^	0.001	< 0.001***	0.001	0.001
	Experienced VCAC (no)^3^	1.361	0.037*	0.084	2.639
	VCAC is/was taken seriously in CAC^#^	0.808	< 0.001***	0.476	1.141
	Experience of Child Maltreatment in the family (no)^4^	3.797	< 0.001***	2.997	4.598

CI = Confidence Interval; ^#^ Continuous Variable * Significant at the 5% level; ** Significant at the 1% level; *** Significant at the 0.1% level; ^a^ assessed via the WHO-5; ^1^ Reference category: age at the date of the survey > 60; ^2^ Reference category: male; ^**3**^ Reference category: experienced VCAC; ^**4**^ Reference category: experienced child maltreatment in family

## Discussion

Using data from a representative sample of the German general population, this study determined the prevalence rates of emotional, physical and sexual VCAC in Germany, investigated the frequency of protective measures against VCAC, and showed correlations with the feeling of safety in CACs. 8.42% reported at least one form of VCAC in childhood/adolescence, with awareness of protective activities decreasing with age and increasing with membership in certain clubs. Violence prevention activities in CACs were often perceived as addressing violence, providing opportunities to complain, and providing information about (sexual) violence.

Activity in music, youth, and sports clubs dominate during childhood and adolescence, which underlines the importance of these types of clubs for childhood and adolescent development. Another crucial finding is that adults who were already active in clubs in their childhood/youth were more likely to be involved in volunteer work with children and adolescents. This suggests that early experience in club work increases the likelihood of long-term involvement in volunteer work. Those persons are particularly likely to be involved in music clubs, social and rescue organizations, and church associations. The role of the church and social/rescue CACs as places of long-term engagement could be related to their strong community orientation and the opportunity to be socially useful.

The results of this study reveal that, of the 819 participants cared for in CACs, 8.42% (*n* = 69) reported at least one type of VCAC not differentiated for adults or peers as perpetrators. This is consistent with existing findings in the literature that indicate that violence is a significant problem in volunteer organizations. One study indicated that children and adolescents in sports clubs are at increased risk of physical and emotional violence and sexual abuse [35]. Prevalence rates vary, but similar studies have documented comparable percentages of violent incidents in youth organizations [36, 37]. Compared with the prevalence rates of child maltreatment for the whole German population, which are approximately 31%, the prevalence of VCAC is lower [12].

Notably, girls were more likely to experience all the surveyed forms of VCAC (multiple victimization). This difference is most evident in sexual VCAC, for which only one boy but six girls had been affected. This finding is consistent with those of studies showing that girls are more vulnerable to sexual violence and multiple types of abuse [38, 39]. This can be attributed to gender-specific power relations, social norms, and role models. Girls are often perceived as weaker and more vulnerable, which perpetrators can exploit [40–42]. The high prevalence of participants who had experienced more than one form of VCAC indicates a possible comorbidity between the forms, which has already been confirmed in other studies of child maltreatment [12]. Prevention measures must therefore be comprehensive and not target only a single form of VCAC.

In addition, the experience of child maltreatment in the family significantly affects the perception of one’s own well-being later in life. These findings are consistent with those found in the existing literature, which shows that violence in childhood and adolescence can have profound somatic and psychological consequences that negatively affect overall well-being [43]. It is important to note that family violence is the most common form of violence and must therefore also be considered a strong predictor of well-being regardless of the experience of VCAC (Unabhängige Beauftragte für Fragen des sexuellen Kindesmissbrauchs, n.d.). Interestingly, however, it also shows that subjective well-being increases when respondents feel that VCAC is taken seriously in the CAC. This underlines the importance of a strong institutional response to VCAC. When children and adolescents see that their concerns are taken seriously and action is taken to protect them, this can have a positive impact on their well-being both now and later in life and can therefore be seen as a protective effect. It is therefore not enough to simply prevent violence; a culture of respect and support must also be created in which children and adolescents feel safe and valued. Volunteers should therefore be specifically trained in developing skills that enable them to build respectful, supportive, and non-violent relationships with children and adolescents and improve their ability to respond to VCAC. Promoting respectful interaction could therefore not only reduce the immediate risk of VCAC but also have long-term positive effects on the social climate and the development of children and adolescents.

With respect to prevention strategies in CACs, respondents who were active in CACs as children and/or adolescents most frequently perceived the discussion of violence, opportunities to complain and information on (sexual) violence as elements of prevention. On the other hand, adults are particularly aware of the participation of children and adolescents, codes of conduct and opportunities to lodge complaints. It is striking that only 8.91% of the children and adolescents perceived opportunities for participation. This could indicate a communication gap or a lack of child-friendly presentations of information and opportunities for participation.

Furthermore, the results indicate that only 7.5% of respondents who were active in CACs as children and/or adolescents, and 10.7% of adults, reported the development of protection measures in their organizations. Prevention programs against (sexual) violence were perceived by only 5% of children and adolescents and 15% of adults. These figures are low compared with the recommendations of the WHO, which emphasize that preventive programs and training should be accessible and known to all stakeholders [44]. The low awareness of training (4.38%) and intervention plans (5.52%) among adults indicates a possible deficit in education and awareness. This is consistent with the findings of other surveys that have shown that although some CACs are already making efforts to protect children and adolescents from violence, not all CACs are currently involved in this safeguarding progress [19, 25]. However, in other areas in which work is carried out with children and adolescents in Germany, such as education, parenting and health, there is an increasing awareness of safeguarding measures and better protection of the children and adolescents in care. In these areas, however, comprehensive measures are often still lacking, although obligations already seem to be taking effect here that do not currently exist in such a comprehensive form in the voluntary sector.

A linear regression shows that age at the time of the interview, membership in a certain CAC, and experience of VCAC are significant predictors of perceptions of the number of safeguarding elements. Older participants perceived fewer protective measures, which may be because their membership in CACs dated back longer, possibly at a time when awareness of VCAC was much lower and no appropriate protective measures were taken. Membership in music, youth political or social/rescue-oriented clubs increased perceptions of protection measures, which could also indicate that there is a greater awareness of the issue of protection against violence in these CACs. This suggests that the respondents in these CACs had positive and supportive experiences as children/adolescents that made them aware of protective measures and fostered a sense of safety, whereas the experience of violence in CACs decreased their perceptions.

Historical and structural differences can be assumed for VCAC in Germany, caused by the inner German division in the former German Democratic Republic (GDR) and the Federal Republic of Germany (FRG) from 1949 to 1990 [45, 46]. In the GDR, youth activities were largely controlled by the state, with organizations such as the Pioneer Organization and the Free German Youth playing a central role. These state-controlled offerings, supplemented by various sports programs, were formative for most young people. In contrast, church activities played a subordinate role in the GDR because the state pursued a restrictive religious policy that severely limited church activities [47]. In comparison, there was a greater variety of youth activities in the FRG, including a strong presence of church organizations [48], where reports of maltreatment and violence have increasingly come to public attention recently [31]. In the FRG, involvement in associations was predominantly voluntary but was also directed by, for example, church institutions. Further analysis and research are needed to examine the differences in VCAC between the two countries. To be able to draw conclusions outside the German population, it is also necessary to discuss the influence of cultural factors on the frequency of volunteering and the experience of violence and protective measures in different cultural contexts. For example, countries with a long tradition of volunteering may have higher rates of participation in CACs than cultures where volunteering is less established, which can lead to different meanings of CACs as spaces for children and adolescents to grow up. Furthermore, in some cultures there is a higher sensitivity to violence against children and adolescents. The social acceptance or tabooing of violence influences whether those affected report their experiences at all [49]. In countries where violence or misuse of power is less frequently discussed, such safeguarding measures may be less well known or enforced. An international comparison could help to better understand and interpret these effects.

The lower prevalence of violence in CACs compared with the overall prevalence of child maltreatment in Germany and the potential of CACs as supervised yet creative spaces where children can explore, take responsibility, and learn self-determination show how important CACs are for children and adolescents. It also highlights how essential it is to protect children and adolescents from violence and to support those affected by CACs through safeguarding measures. In Germany, an online platform with training courses to teach and sensitize volunteers concerning VCAC and the development of safeguarding measures in CACs has raised awareness for VCAC (https://engagement-schutzkonzepte.elearning-kinderschutz.de/) [50]. However, there are still enough areas of volunteer work in which no awareness-raising or training on the topic takes place and volunteers are not reached, which makes protecting children and adolescents more difficult and increases the risk of violence. Safeguarding measures should therefore become mandatory for CACs to provide children and adolescents with the best possible protection, and volunteers should be made more aware of the issue of VCAC.

### Limitations & strengths

This study’s major strength is the population-based composition character and the size of the sub-sample, which provides, for the first time, data on the prevalence of VCAC in Germany. Considering the lack of previous studies in this field, this study contributes important knowledge that will facilitate a better understanding of VCAC and its consequences. However, the main limitation is the cross-sectional design. The outcomes of VCAC were derived from retrospective self-reports, which may result in recall bias and, consequently, underreporting. Furthermore, due to the age of the respondents, it is difficult to draw generalizable conclusions about the current state of VCAC and safeguarding measures in CAC, as the respondents’ experiences often date back several years/decades. In addition, respondents may not have classified their experiences as VCAC because they may have lacked an awareness of what VCAC comprises. The extent of the recall bias is unknown because no data were collected on how long-ago membership in the CACs took place. In addition, the different historical contexts of the GDR and FRG were not considered in activity in CACs. In the GDR in particular, there may have been systematic underreporting of incidents of violence because these issues were rarely discussed in public and may have been suppressed by the state (Sachse et al., 2017). The survey approach cannot consider the recent heightened awareness of VCAC. In addition, the number of respondents who reported on VCAC was relatively low, at 69. The causality cannot be inferred because this is an observational study. The items utilized for the survey of VCAC were not included in the validated questionnaire because of a dearth of instruments that assess forms of VCAC; however, these instruments have been oriented to survey methods that address experiences of maltreatment in general. The high number of respondents in the category “other CAC” shows that many of the respondents were unsure which category they should assign themselves to, even though examples were given. This limitation must be considered in the calculations where the type of CACs was included.

## Conclusions

Overall, the results of this study underline the importance of club work for children and adolescents. They indicate that CACs that successfully retain children and adolescents play an important role in promoting social engagement and community participation in current and later life. There is an urgent need to strengthen systematic and comprehensive protective measures in CACs because the prevalence of VCAC is high. These measures could include strengthening supervision and protection mechanisms, training volunteers in recognizing and reporting VCAC, and promoting a culture of awareness and respect for children and adolescents. Safeguarding measures, which are now mandatory in medical and educational institutions in Germany and include, among others, risk analysis, further training, codes of conduct, voluntary commitments and obtaining an extended certificate of good conduct, should also be required by law in CACs. The participation of volunteers in the development and implementation of safeguarding measures is also crucial to ensure that these measures are practical and effective (UBSKM. 2013). In this way, a culture of mindfulness and the respectful treatment of proximity and distance can be promoted (DBK, 2015). Further research on this topic and practical interventions are needed to gain a better understanding of the causes and effects of VCAC and to develop evidence-based interventions. A comparison with the literature revealed that the prevalence rates and gender differences reported here are largely consistent with those reported in previous studies, underscoring the relevance and urgency of this issue. There is also a need for improvement in the communication and implementation of safeguarding measures in CACs. The discrepancy between the perceptions of children and adults, as well as the low awareness of prevention and intervention programs, underscores the need for targeted measures to raise awareness and educate all those who are active in a CAC.

## Electronic supplementary material

Below is the link to the electronic supplementary material.

Supplementary Material 1: Additional file 1 - Questionnaire on sociodemographic data and experiences and protection of violence against children and adolescents in voluntary clubs from a representative survey of the German population between 10/2023 and 3/2024 (translation from German).

## Data Availability

The datasets used and/or analyzed during the current study are available from the corresponding author on reasonable request.

## Abbreviations

CAC: Child and Adolescent Club
CDC: Centers for Disease Control and Prevention
FRG: Federal Republic of Germany
GDR: German Democratic Republic
VCAC: Violence against children and adolescents in clubs
WHO: World Health Organization
WHO-5: World Health Organization-Five Well-Being Index
