# Supplementary material for: Prevalence and prevention of violence against children and adolescents in volunteer work: analysis of a sub-sample from a representative survey of Germany
Source: BMC Public Health. 2025 May 27;25:1951. doi: 10.1186/s12889-025-23038-y (PMC12107928; doi:10.1186/s12889-025-23038-y)
Supplement: Supplementary file 1 — Supplementary Material 1: Additional file 1 - Questionnaire on sociodemographic data and experiences and protection of violence against children and adolescents in voluntary clubs from a representative survey of the German population between 10/2023 and 3/2024 (translation from German). [file 12889_2025_23038_MOESM1_ESM.pdf]

Additional file 1

Questionnaire on sociodemographic data and experiences and protection of violence against children and adolescents in voluntary clubs from a representative survey of the German population between 10/2023 and 3/2024 (translation from German)

Sociodemographic Data

S1	*Your gender*	Male	☐
		female	☐
		diverse	☐

S2	*When were you born?*		
		**Month of birth (e.g. 09):**	☐☐
	(Please tell only the month and year of birth).		
		**Year of birth:**	☐☐☐☐

S4	*What is your marital status?*	I am married and live with my spouse	☐	➔ continue with **Question S5**
		I am married and live separately from my spouse	☐	➔ continue with **Question S4a**
		I am unmarried	☐	
		I am divorced	☐	
		I am widowed	☐	

S4a	*Are you in a stable partnership?*	Yes	☐
		No	☐

S5	*How many people live in your household all the time - meaning people who sleep and eat here, including yourself?*	☐☐	**People**

Additional file 1

Questionnaire on sociodemographic data and experiences and protection of violence against children and adolescents in voluntary clubs from a representative survey of the German population between 10/2023 and 3/2024 (translation from German)

Sa	*How many of them are children* ***under the age of 16****?*<br>(Enter the number of persons or "0" in each case).	Children under the age of 16	☐☐ People

S6	*What nationality do you have?*	German	☐
		other, which: ____________________________	☐

S7	*Which religious community or denomination do you belong to?*	Protestant	☐
		Catholic	☐
		Muslim	☐
		other (e.g. Jewish, Buddhist, Hindu)	☐
		no denomination	☐

Additional file 1

Questionnaire on sociodemographic data and experiences and protection of violence against children and adolescents in voluntary clubs from a representative survey of the German population between 10/2023 and 3/2024 (translation from German)

S8	*If there were a general election next Sunday, would you go to the polls?*	Yes	☐	➔ continue with **Question S9**
		No	☐	➔ continue with **Question S10**
		Don't know	☐	
		I am not eligible to vote	☐	
		not specified	☐	

S9	*And which party would you vote for?*<br><br>(Do NOT read out the answer levels! Only one answer!)	CDU	☐
		CSU	☐
		SPD	☐
		AfD	☐
		FDP	☐
		Die Linke	☐
		Bündnis 90/Die Grünen	☐
		other party	☐
		don't know	☐
		vote invalid	☐
		not specified	☐

Additional file 1

Questionnaire on sociodemographic data and experiences and protection of violence against children and adolescents in voluntary clubs from a representative survey of the German population between 10/2023 and 3/2024 (translation from German)

S10	*What school-leaving qualification do you have?*	I left school without a secondary school leaving certificate	☐
		I have a secondary school leaving certificate (or previous 8-grade school).................................	☐
		I have a secondary school leaving certificate	☐
		I have completed the 10th grade of polytechnic secondary school..	☐
		I have a technical college degree (without recognition as a university of applied sciences degree)	☐
		I have a general or subject-restricted higher education entrance qualification (Gymnasium or EOS) without having completed a higher education/university degree	☐
		I have a university/college or university of applied sciences degree (or university of applied sciences degree with recognition as a university of applied sciences degree)...	☐
		I have a different school-leaving qualification, namely:_______________________________________	☐
		I am a pupil at a general education school	☐

Additional file 1

Questionnaire on sociodemographic data and experiences and protection of violence against children and adolescents in voluntary clubs from a representative survey of the German population between 10/2023 and 3/2024 (translation from German)

S11	*What on this list applies to you?*	Full-time employment with a weekly working time of 35 hours or more	☐
		Part-time employment with a weekly working time of 15 to 34 hours	☐
		Part-time or hourly employed with a weekly working time of less than 15 hours	☐
		Federal voluntary service, maternity/parental leave or other leave of absence	☐
		currently unemployed/ in 0 short-time work	☐
		Pensioner/early retirement	☐
		not working (e.g. housewife/househusband)	☐
		in vocational training (including technical schools for industrial professions)	☐
		in school education (including university, college)	☐

S12	*How often have you been unemployed, including current unemployment?*	☐☐	**Paint**

S14	*Please take a look at this list of income groups.*<br>*What is* ***your own*** *income?*<br>*I mean, what is your net monthly income after deduction of tax and social security?*<br>*All you need to do is to tell the number.*<br>(Submit list 14)	No. Income group:	☐☐
		No personal income	☐

Additional file 1

Questionnaire on sociodemographic data and experiences and protection of violence against children and adolescents in voluntary clubs from a representative survey of the German population between 10/2023 and 3/2024 (translation from German)

S15	*And in which group would you classify your household in terms of* ***total*** *monthly net income?*<br>*I mean the sum resulting from wages, salary, income from self-employment, pension or annuity, in each case after deduction of taxes and social security contributions.*<br>*and social security contributions. Please also include income from public benefits, income from renting, leasing, housing benefit, child benefit and other income.*<br><br>(Submit list 14)	No. Income group: ☐☐<br><br>Please enter the corresponding number.

Additional file 1

Questionnaire on sociodemographic data and experiences and protection of violence against children and adolescents in voluntary clubs from a representative survey of the German population between 10/2023 and 3/2024 (translation from German)

Scientists under the direction of the University of Leipzig would like to use the results of this study to verify various scientific/medical findings. It is about you personally, your experiences, your behavior, your sensitivities. Please answer the following questions by ticking the answer that best applies to you. There are no "right" or "wrong" answers. Your information will be treated in strict confidence. Please read the information in the enclosed data protection sheet.

Experiences and protection of violence against children and adolescents in voluntary clubs

G12.	Are you or have you been an active member of one or more associations whose purpose is to work with children and/or adolescent?<br>(e.g. sports club, music club, Green Youth, Free German Youth, Young European Supporters, Youth Ring, rural youth, German Red Cross, fire department, altar servers)<br>*Active membership means that you are actively involved in fulfilling the purpose of the association. Passive members only support the association through their membership and the membership fee.*	yes, only in adulthood	☐	
		yes, only as a child/adolescent	☐	
		yes, both as a child/adolescent and as an adult	☐	
		no	☐	➔ continue with question G19

G13.	In which area(s) are/were you an active member of one or more associations whose purpose includes working with children and/or adolescent?<br><br>*(multiple answers possible)*	Sports club	☐
		Music club	☐
		Youth political association (e.g. Young Socialists, Green Youth, Free German Youth, Young European Federalists)	☐
		Social association (e.g. youth ring, rural youth)	☐
		Social/rescue association (e.g. DRK, fire department) ...	☐
		Church association (e.g. altar servers)	☐
		Other Cliub	☐

Additional file 1

Questionnaire on sociodemographic data and experiences and protection of violence against children and adolescents in voluntary clubs from a representative survey of the German population between 10/2023 and 3/2024 (translation from German)

G14.	*If G12:*<br>*"yes, only as a child/adolescent" or "yes, both as a child/adolescent and as an adult"*<br><br>Please indicate below which measures to protect children/adolescent from (sexual) violence you have encountered **during your membership in the club as a child/adolescent.** This includes sexual as well as other violence in the club.<br><br>*(multiple answers possible)*	Addressing the topic of (sexual) violence by youth leaders/trainers	☐
		Implementation of prevention programs on (sexual) violence	☐
		Complaints options (e.g. complaint box)........	☐
		Defined code of conduct/declaration of commitment/code of honor for dealing with each other in the club	☐
		Co-determination/participation opportunities for children/adolescents (e.g. children's conferences, participation projects)	☐
		Addressing misconduct in the context of (sexual) violence by youth leaders/trainers	☐
		Punishment of misconduct in the context of (sexual) violence by youth leaders/trainers........	☐
		Naming a person who can be approached if you experience (sexual) violence, have a suspicion or an uneasy gut feeling can be addressed........	☐
		Information (e.g. posters, notices, flyers) on (sexual) violence on the club's premises	☐
		Development of safeguarding measures for the association........	☐
		Other	☐

G15.	*If G12:*<br>*"yes, only as a child/adolescent" or "yes, both as a child/adolescent and as an adult"*<br><br>Have you ever experienced violence/maltreatment as a child/adolescent when you were a member of a club?	Yes	☐	
		No	☐	➔ continue with question G17

G16.	What kind of violence have you experienced?<br><br>*(multiple answers possible)*	Emotional maltreatment/violence	☐
		Physical maltreatment/violence........	☐
		Sexual maltreatment/violence	☐

Additional file 1

Questionnaire on sociodemographic data and experiences and protection of violence against children and adolescents in voluntary clubs from a representative survey of the German population between 10/2023 and 3/2024 (translation from German)

If G12:
"yes, only as a child/adolescent" or
"yes, both as a child/adolescent and as an adult"

How strongly do you agree with the following statements **regard to your club membership(s) as a child/adolescent**

G17.		do not agree at all					do agree completely
		1	2	3	4	5	6
01	I felt/feel safe from (sexual) violence in my club.	☐	☐	☐	☐	☐	☐
02	The youth leaders/trainers all children/adolescents with respect.	☐	☐	☐	☐	☐	☐
03	The children/adolescents treat each other respectfully.	☐	☐	☐	☐	☐	☐
04	Cases of (sexual) violence are/were taken seriously in my club.	☐	☐	☐	☐	☐	☐
05	Violence of any kind is/was not tolerated in my club.	☐	☐	☐	☐	☐	☐

Additional file 1

Questionnaire on sociodemographic data and experiences and protection of violence against children and adolescents in voluntary clubs from a representative survey of the German population between 10/2023 and 3/2024 (translation from German)

G18.	*If G12: "yes, only in adulthood" or "yes, both as a child/adolescent and as an adult"*<br><br>Please indicate below which measures to protect children/adolescent from (sexual) violence you have encountered **in the course of your membership of the association as an adult**. This includes sexual but also other violence in the club<br><br>*(multiple answers possible)*	Addressing the topic of (sexual) violence by the Executive Board	☐
		Implementation of prevention programs on (sexual) violence...	☐
		Complaints options (e.g. complaint box)	☐
		Defined code of conduct/declaration of commitment/code of honor for dealing with each other..	☐
		Opportunities for co-determination/participation for all groups in the club (children/adolescents, parents, youth leaders/trainers) e.g. children's conferences, participation projects	☐
		Addressing misconduct in the context of (sexual) violence by the Executive Board	☐
		Punishment of misconduct in the context of (sexual) violence by the Executive Board	☐
		Designate a contact person who can be approached if (sexual) violence is experienced, suspected or if you have a bad gut feeling..	☐
		Information (e.g. posters, notices, flyers) on (sexual) violence on the club's premises.................................	☐
		Development of safeguarding measures	☐
		(Sexual) educational concept for working with children/adolescents	☐
		Requesting an extended certificate of good conduct from all those working in the club	☐
		Further training on (sexual) violence in the context of club activities	☐
		Intervention plan for dealing with (sexual) violence....	☐
		Others	☐

Additional file 1

Questionnaire on sociodemographic data and experiences and protection of violence against children and adolescents in voluntary clubs from a representative survey of the German population between 10/2023 and 3/2024 (translation from German)

List 14

Group No	Net monthly income
1	up to under 500 EUR
2	500 to under 650 EUR
3	650 to under 750 EUR
4	750 to under 900 EUR
5	900 to under EUR 1,000
6	1,000 to under EUR 1,150
7	1,150 to under EUR 1,250
8	1,250 to under EUR 1,500
9	1,500 to under EUR 2,000
10	2,000 to under EUR 2,500
11	2,500 to under EUR 3,500
12	EUR 3,500 to less than EUR 5,000
13	EUR 5,000 and more
14	No personal income
